# Beneficial Role of Heat‐Treated *Lactobacillus sakei* HS‐1 on Growth Performance, Nutritional Status and Gut Microbiota in Weaned Piglets

**DOI:** 10.1111/jpn.14056

**Published:** 2024-10-16

**Authors:** Kazuki Matsubara, Junyou Li, Yuriko Enomoto, Tomotsugu Takahashi, Min Ma, Ryo Ninomiya, Daiji Kazami, Kozue Miura, Kazuhiro Hirayama

**Affiliations:** ^1^ Laboratory of Veterinary Public Health, Graduate School of Agricultural and Life Sciences The University of Tokyo Tokyo Japan; ^2^ Animal Resource Science Center, Graduate School of Agricultural and Life Sciences The University of Tokyo Ibaraki Japan; ^3^ Daiwa Pharmaceutical Co. Ltd. Tokyo Japan; ^4^ Kazami Food Science Tochigi Japan; ^5^ Research Center for Food Safety, Graduate School of Agricultural and Life Sciences The University of Tokyo Tokyo Japan

**Keywords:** amino acid, *Lactobacillus sakei* HS‐1, microbiota, short‐chain fatty acid, weaned piglet

## Abstract

In the swine industry, there is a strong need to replace an antibiotic growth promoter (AGP) used as feed additives in weaned piglets to enhance nutrient utilization in their diets and improve growth performance. *Lactobacillus sakei* HS‐1 strain is a microbial preparation isolated from pickles. The study aim is to investigate the effectiveness of heat‐treated *L. sakei* HS‐1 strain (HT‐LS) as a growth promoter in weaned piglets compared to colistin (CS), a widely used AGP. Eighteen crossbred weaned piglets (Landrace × Yorkshire × Duroc) of 21 days (average body weight [BW]: 7.06 ± 0.59 kg) were divided into three groups: fed the control diet (CT group), fed a diet supplemented with 30 ppm colistin sulphate (CS group), fed a diet supplemented with HT‐LS at a concentration of 2.0 × 10^5^ cells/g (LS group) until 49 days. The results indicated that LS group exhibited significantly higher average daily gain (*p* < 0.05) and higher BW (*p* < 0.1) compared with CT group, even higher than CS group. CS group showed higher growth performance compared to CT group but the differences were not statistically significant. In addition, LS group had higher (*p* < 0.05) or tended to higher (*p* < 0.1) concentrations of several plasma amino acids than the other two groups at 35 and 49 days. Faecal acetate concentration was higher (*p* < 0.1) in LS group than in CT group at 35 days. Blood immunoglobulin G concentration in LS group was significantly lower (*p* < 0.05) than in CT group at 35 and 49 days, and blood immunoglobulin A tended to be lower (*p* < 0.1) at 35 days than in CT group. LS group showed an increased abundance of g_Prevotella 7, g_Streptococcus and g_Lactobacillus (linear discriminant analysis [LDA] score ≥ 2.0). Predictive metagenomic analysis revealed an enrichment of the mixed acid fermentation pathway (LDA score ≥ 2.0). Furthermore, several gut microbes exhibited correlations with plasma amino acids (*p* < 0.01) and short‐chain fatty acids in faeces (*p* < 0.01). These findings demonstrate that HT‐LS improves the growth performance of weaned piglets by enhancing the efficient utilization of nutrients through gut microbiota modification.

**Abbreviations**
ADFI: average daily feed intake
ADG: average daily gain
AGP: antibiotic growth promoter
ASV: amplicon sequence variant
BW: body weight
CS: colistin sulphate
CT: control
*E. coli*
: *Escherichia coli*
ELISA: enzyme‐linked immunosorbent assay
FCR: feed conversion ratio
HSD: honestly significant difference
HT‐LS: heat‐treated *Lactobacillus sakei* HS‐1 strain
IgA: immunoglobulin A
IgG: immunoglobulin G
LDA: linear discriminant analysis
LEfSe: Linear discriminant analysis Effect Size
LS: *Lactobacillus sakei* HS‐1 strain
*L. sakei*
: *Lactobacillus sakei*
PBS: phosphate‐buffered saline
QIIME2: Quantitative Insight Into Microbial Ecology 2
SCFAs: short‐chain fatty acids
sIgA: secretory immunoglobulin A

## 1 Introduction

During the weaning stage, piglets experience significant stress due to a new herd environment, rapid changes in feed components, and the loss of passive immunity. This susceptibility to infection greatly impacts their subsequent growth. As such, the weaning stage holds the utmost importance in swine production. An antibiotic growth promoter (AGP) is often employed to enhance nutrient utilization in feed, potentially by preventing infectious diseases (Rabelo‐Ruiz et al. 2021). However, the emergence of antimicrobial resistance necessitates the development of alternative approaches. Probiotics, defined as live microorganisms with beneficial effects on humans and/or animals through improved gut microbiota balance. *Lactobacillus*, a genus of lactic acid bacteria, in particular, is expected to be as a growth promoter by regulating immune system, improving growth performance, feed conversion efficiency, nutrient utilization, gut microbiota, and gut health in pigs (Dowarah, Verma, and Agarwal 2017). *Lactobacillus sakei* is widely used as a starter for food fermentation (Zagorec and Champomier‐Vergès 2017), and it has also been reported that feeding heat‐treated *L. sakei* HS‐1 (HT‐LS), derived from a strain isolated from kimchi, a traditional Korean fermented food, to newborn calves and broilers improves growth performance (Khonyoung and Yamauchi 2019; Sasazaki et al. 2020, 2023). Furthermore, HT‐LS markedly enhances mucosal immunity and resistance to pathogens in mice (Ghoneum and Abdulmalek 2021). Based on the above reports, HT‐LS can be a candidate for growth promoter alternative to antibiotics. However, no studies, to our knowledge, have explored its effects on swine production.

The unique characteristics of the HT‐LS is that the preparation is heat killed. Administration of heat‐treated probiotics, referred as postbiotics, to weaned piglets has demonstrated diarrhoea reduction and improved growth performance (Kang et al. 2021). It has also been reported that a heat‐treated and dried cell preparation of *Enterococcus faecalis* ameliorated intestinal villi atrophy (Tsukahara et al. 2011). Heat‐treated bacterial preparation may sometimes be superior to probiotics because of their stability and safety (Zhong et al. 2022).

The gut microbiota metabolizes short‐chain fatty acids (SCFAs) and amino acids (Portune et al. 2016; Hu et al. 2023), and one of the important mechanisms of the effects of probiotics and postbiotics is the effects on the composition and metabolism of the gut microbiota (Zhu et al. 2020). So, we hypothesized that HT‐LS also improves nutritional performance of pigs.

The objective of this study is to investigate the efficacy of HT‐LS in comparison to colistin sulphate (CS), a commonly used growth‐enhancing antibiotic agent in swine production (Andrade et al. 2020; Rhouma, Madec, and Laxminarayan 2023), especially for weaned piglets, and to discuss its mechanism from a gut microbiota and nutritional perspectives. Therefore, weaned piglets were fed diets containing HT‐LS or CS, and growth performance and blood immunoglobulins, as an indicator of immune function, as well as the composition of gut microbiota were examined. Amino acids are essential nutrients for piglet, and blood amino acid concentrations are related to growth performance (Trevisi et al. 2009). Faecal SCFAs are utilized as energy for pigs and associated with weight gain (Zhou et al. 2019). Hence, in this study, blood amino acids and SCFAs in faeces were also examined as indicators of nutritional status.

## 2 Materials and Methods

### 2.1 Animals and Experimental Design

The experiment was conducted at the Animal Resource Science Center of the University of Tokyo (Ibaraki, Japan), and approved by the Animal Care and Use Committee of the University of Tokyo (P21‐031). A total of 18 crossbred healthy weaned piglets [(Landrace × Yorkshire) × Duroc] derived from two sows raised exclusively on each sow's milk in a general pig farm, with a mean body weight (BW) of 7.06 ± 0.59 kg at 21 days of age, were transported to the Animal Resource Science Center of the University of Tokyo, and randomly assigned to three groups of six piglets (Table 1). The experimental facility had the semi‐closed type animal housing, and was maintained at approximately 20°C degrees, and the average humidity was 69% under natural photoperiods, which was a light phase of 12 h and a dark phase of 12 h. The piglets from each sow were distributed evenly to each group. Control (CT) group received an early‐stage diet (Japan Scientific Feeds Association, Tokyo, Japan) which meets the National Research Council standards (NRC 2012) without any antibiotics (Supporting Information S1: Table S1). CS group received the same early‐stage diet supplemented with 30 ppm CS (Meiji Seika Pharma. Co. Ltd., Tokyo, Japan), and LS group received an early‐stage diet supplemented with HT‐LS (Daiwa Pharmaceutical Co. Ltd., Tokyo, Japan) at a rate of 2.0 × 10^5^ cells/g. The piglets had unrestricted access to both the experimental diets and drinking water. Weekly records were kept of the total feed consumption in each pen. BW of the piglets was individually measured at 21, 28, 35, 42, and 49 days of age. Finally, the piglets were killed at 49 days of age, after 28 days of feeding of the experimental diet.

**Table 1:** Growth performance of weaned piglets throughout experiment period.

	Initial BW, kg	Final BW, kg	ADG, kg	ADFI, kg	FCR
CT group	7.06 ± 0.14	15.02 ± 0.97	0.28 ± 0.03	0.48	1.69
CS group	7.07 ± 0.26	16.73 ± 0.78	0.34 ± 0.02	0.49	1.42
LS group	7.06 ± 0.20	18.31 ± 1.09†	0.40 ± 0.03*	0.61	1.52

*Note*: The mean and SEM are indicated. *n* = 6 per group.

Abbreviations: ADFI, average daily feed intake; ADG, average daily gain; BW, body weight; CS group, weaned piglets supplemented with early‐stage diet added 30 ppm colistin sulphate; CT group, weaned piglets supplemented with early‐stage diet; FCR, feed conversion ratio; LS group, weaned piglets supplemented with early‐stage diet added heat‐treated *Lactobacillus sakei* HS‐1 strain at a rate of 2.0 × 10^5^ cells/g.

* *p* < 0.05 compared to CT group by Tukey's HSD test.

^†^ *p* < 0.1 compared to CT group by Tukey's HSD test.

### 2.2 Sample Collection

Blood was collected weekly from the jugular vein immediately after weighting with a syringe containing EDTA‐Na. The collected blood was centrifuged for 10 min (3000 rpm) at 4°C to obtain plasma. The plasma was subsequently stored at −80°C until analysis. Fresh faeces were collected individually from the anus by stimulating the rectum using a sterile swab after weighting, immediately frozen, and stored at −80°C for subsequent analysis. Blood and faecal samples were collected at the same time each week in the morning. At the end of the experiment, piglets were slaughtered by electrocution following the administration of Thiamylal Sodium (ISOZOL for Injection 0.5 g, Nichi‐Iko Pharmaceutical Co. Ltd., Toyama, Japan). After slaughter, the intestinal tract was extracted and specific segments (duodenum, jejunum, ileum, colon) were defined as follows: duodenum −2 cm from the pylorus region; jejunum −50 cm from duodenum; ileum −50 cm from caecum to cranial side; colon −50 cm from caecum to caudal side. The segments, approximately 6 cm in length, were collected using sterilized instruments and flushed the contents thoroughly with sterile phosphate‐buffered saline (PBS), and cut open. Then mucosal layer was scraped and immediately stored at −80°C until required for analysis.

### 2.3 Amino Acids Analysis

Plasma was mixed with a three‐fold vol/wt. acetonitrile, vortexed for 10 s, centrifuged at 4°C, 6000 rpm for 10 min, and filtered through a PVDF 0.45 μm membrane (Merck Millipore, Massachusetts, USA) to remove protein. A 100 μL of the processed samples was diluted with 350 μL of 100 mM sodium phosphate buffer. Amino acids were derivatized with 4‐fluoro‐7‐nitro‐2,1,3‐benzoxadiazole (NBD‐F, Tokyo Chemical Industry Co. Ltd., Tokyo, Japan) by adding 50 μL of 50 mM NBD‐F (dissolved in acetonitrile), heated at 60°C for 1 min, and cooled on ice. After adding 300 μL of 5 mM HCL, 2 μl of the reaction mixture was injected into an HPLC system (Chromaster, Hitachi, Tokyo, Japan) connected to an ODS column (CAPCELL PAK C18 MGII S‐5, 4.6 mm I.D. × 150 mm, OSAKA SODA Co. Ltd., Osaka, Japan) with 10 mmol/L citrate buffer with 75 mmol/L NaClO_4_/CH_3_CN = 50/50 at a flow rate of 0.4 mL/min. Fluorescence detection of the NBD‐amino acids was performed at 530 nm with excitation at 480 nm. The concentrations of amino acids were determined using amino acid standards (FUJIFILM Wako Pure Chemical Corporation, Osaka, Japan).

### 2.4 SCFAs Analysis

Approximately 50 mg of faeces were mixed with a 20‐fold vol/wt. 6% HClO_4_, vortexed thoroughly, and centrifuged at 4°C, 14,800 rpm for 10 min. The supernatant was subjected to another centrifugation under the same conditions and filtered using a PVDF 0.45 μm membrane. A 50 μL aliquot of the processed samples was injected into an HPLC system connected to a size exclusion chromatography column (GL‐C610H‐S, 7.8 mm I.D. × 300 mm, Hitachi Chemical Co. Ltd., Tokyo, Japan) with 3 mM HClO_4_ as the mobile phase, flowing at a flow rate of 0.5 mL/min. For the detection of SCFAs, the analysis was conducted at 440 nm after the addition of 0.1 mM BTB‐15 mM Na_2_HPO_4_−2 mM NaOH (pH = 9.6).

### 2.5 Immunoglobulins Analysis of Plasma and Intestinal Mucosa by Elisa Kit

Immunoglobulins in plasma samples collected at 21, 35, and 49 days were quantified using a sandwich immunoglobulin A (IgA) pig enzyme‐linked immunosorbent assay (ELISA) kit (Pig IgA ELISA Kit, Bethyl Laboratories, Texas, USA), and immunoglobulin G (IgG) ELISA Kit (Pig IgG ELISA Kit, Bethyl Laboratories). Additionally, the levels of duodenal, jejunal, and ileal secretory IgA (sIgA) were assessed using an ELISA kit (sIgA ELISA Kit, Cusabio Biotech Co. Ltd., Wuhan, China). All ELISA protocols were performed by the manufacturer's instructions.

### 2.6 Bacterial DNA Extraction and Purification

16S rRNA gene sequencing was conducted using faecal samples collected 35 and 49 days. Bacterial DNA extraction was performed as a previously described method (Morita et al. 2007). Approximately 0.2 g of faecal samples were suspended in 5 mL of PBS, filtered through a 100 μm cell strainer (Corning, NY, USA), and subsequently centrifuged at 4°C, 9000 × g for 10 min. The pellets were suspended in 20 mL of PBS and centrifuged under the same conditions. The obtained pellets were further resuspended in 1 mL of TE (10 mM Tris‐HCl, 20 mM EDTA) buffer. Subsequently, lysozyme (Sigma‐Aldrich Co. Tokyo, Japan) was added at a final concentration of 30 mg/mL, and the mixture was incubated for 1 h at 37°C with gentle shaking. Following this purified achromopeptidase (FUJIFILM Wako Pure Chemical Corporation) was added at a final concentration of 2000 units/mL, and the suspension was incubated for 1 h at 37°C with gentle shaking successively. Furthermore, sodium dodecyl sulphate (final concentration of 1 mg/mL) and proteinase K (at a final concentration of 1 mg/mL) (FUJIFILM Wako Pure Chemical Corporation) were added to the suspensions, which were then incubated for 1 h at 55°C with gentle shaking. DNA extraction was performed using phenol/chloroform/isoamyl alcohol (25:24:1, NIPPON GENE Co. Ltd., Tokyo, Japan). The extracted DNA was subsequently precipitated with isopropanol and 3 M sodium acetate, washed with 75% ethanol, and completely dissolved in 100 μL of TE buffer on ice. The DNA was treated with DNase‐free RNase (NIPPON GENE Co. Ltd.) solution at a final concentration of 10 μg/mL, incubated 1 h at 37°C with gentle shaking, purified with a 20% PEG6000‐2.5 M NaCl solution (Hampton Research, California, USA), washed with 75% ethanol thoroughly, and dissolved completely in 100 μL of TE buffer on ice.

### 2.7 16S rRNA Gene Amplicon Sequencing

The bacterial 16S rRNA gene was amplified by two‐step tailed PCR using the universal primers 27Fmod (5′‐AGRGTTTGATYMTGGCTCAG‐3′) and 338 R (5′‐TGCTGCCTCCCGTAGGAGT‐3′), which are specific to the V1–V2 hypervariable region of the 16S rRNA gene as described previously (Kim et al. 2013). The DNA concentration after PCR was 17.3 ng/μL (range 11.9−20.2 ng/μL), and the amplification proceeded without issues. The amplicons were purified using AMPure XP (BECKMAN COULTER, California, USA). Purified amplicons were quantified using a Synergy H1 instrument (Bio Tek, Vermont, USA) and the QuantiFluor dsDNA System (Promega, Wisconsin, USA). Subsequently, the quality of the amplicon was assessed using a Fragment Analyzer (Agilent Technologies, California, USA) and the dsDNA 915 Reagent Kit (Agilent Technologies). 16S rRNA gene sequencing was conducted by Bioengineering Lab. Co. Ltd., Tokyo, Japan, utilizing the Illumina MiSeq platform (Illumina, San Diego USA) in conjunction with the MiSeq Reagent Kit v3 (Illumina). This sequencing process generated 2 × 300 paired‐end reads, adhering to the established Illumina protocols.

### 2.8 Bioinformatic Analysis

The analysis of the sequencing data was conducted using Quantitative Insight Into Microbial Ecology 2 (QIIME2 version 2022.8) (Bolyen et al. 2019). Only the demultiplexed (paired‐end) sequences whose beginning reads obtained from amplicon sequencing data using the fastx_barcode_splitter tool in FASTX‐Toolkit (ver. 0.0.14) (http://hannonlab.cshl.edu/fastx_toolkit/) matched exactly the primer sequences used were selected. Primer sequences were removed from the selected reads using fastx_trimer in FASTX‐Toolkit. Sequences with a quality value of less than 20 were then removed using sickle (ver. 1.33) (https://github.com/najoshi/sickle.), and sequences that were less than 130 bases in length and their paired sequences were discarded. Reads were joined using the paired‐end read joining script FLASH (ver. 1.2.11) (Magoč and Salzberg 2011) with a sequence length of 310 bp after joining, a read joining length of 230 bp, and a minimum overlap of 10 bases. The obtained amplicon sequencing data were imported into the QIIME2 package and analysed by the DADA2 pipeline (Callahan et al. 2016) for quality control, after removal of chimeric sequences. On average, 89.7% of reads were retained after quality control (range 81.6%–93.0%). The samples were assigned to the specific taxonomy information from phylum to species against the SILVA 138 database. Furthermore, unclassified taxonomy was identified with the NCBI BLAST method. To estimate species diversity within samples, the sequences were rarefied at 10,000 depths. The following metrics were assessed for α‐diversity, observed_feature_number, Pielou_evenness, and Shannon indice, by the QIIME2 plugin. As for β‐diversity, weighted UniFrac distance was analysed. Functional predictions for the microbiota were generated using PICRUSt2 (Douglas et al. 2020) using the MetaCyc database (https://metacyc.org) (Karp 2002).

### 2.9 Statistical Analysis

The statistical analysis of growth performance, nutrient indices, and immune function involved conducted one‐way analysis of variance (ANOVA), followed by Tukey's HSD (honestly significant difference) test by using JMP Pro 16 software (SAS Institute, Cary, USA). To examine the possible correlations among microbiota, nutrient indices, and ADG was analysed with Spearman correlation. For β‐diversity measurement, similarity was tested using permutational analysis of variance. For taxonomic composition and functional pathway prediction, the Linear discriminant analysis Effect Size (LEfSe) method was utilized (Segata et al. 2011). The analysis was performed through the LEfSe application (Galaxy version 2.0), available at the following website: http://huttenhower.sph.harvard.edu/galaxy/. The correlation heatmap was generated using the Python (version 3.8.13) programming language modules: Pandas, NumPy, Matplotlib, and Seaborn. *p*‐values less than 0.05 were considered to indicate a statistically significant difference, and *p*‐values less than 0.1 were considered to indicate a tendency.

## 3 Result

### 3.1 Growth Performance

**Figure 1:**
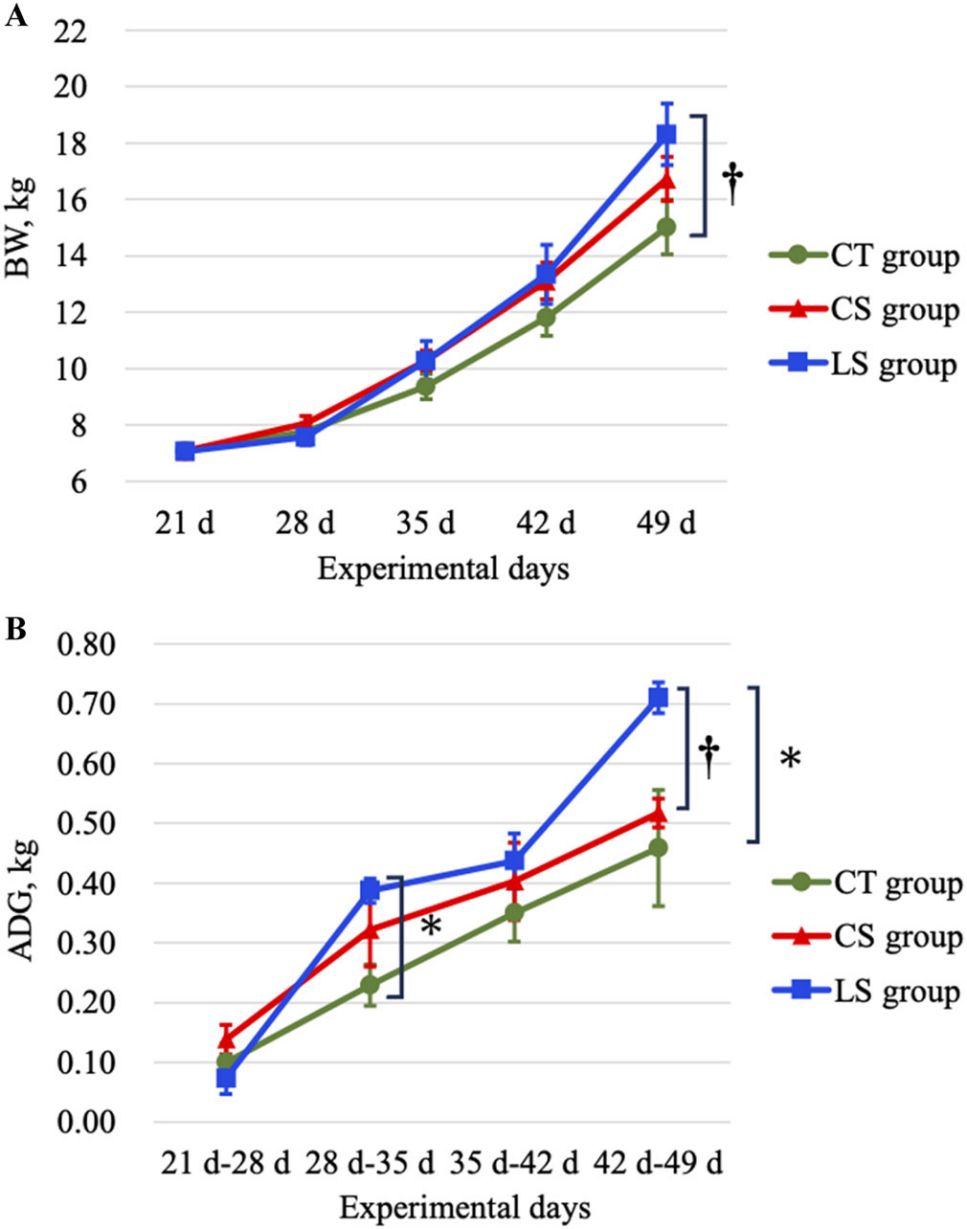
Growth performance of weaned piglets between three groups (*n* = 6) throughout the experimental period. (A) Transition of BW of weaned piglets every week. (B) Transition of ADG of weaned piglets every week. * means *p* < 0.05 compared to CT group by Tukey's HSD test. † means *p* < 0.1 compared to CT group by Tukey's HSD test. [Color figure can be viewed at wileyonlinelibrary.com]

As shown in Figure 1, LS group exhibited a tendency towards higher weight (*p* = 0.068) compared to CT group at the end of the experimental period. Notably, LS group displayed a significantly elevated average daily gain (ADG) at both 35, 49 days and throughout the experiment period (*p* < 0.05) in comparison to CT group, as shown in Figure 1B and Table 1. At 49 days of experiment, ADG of LS group tended to be even better than that of CS group (*p* = 0.089). Although CS group also showed better growth performance, the differences against CT group were not significant. Since all piglets within the same group were housed together, individual feed intake could not be assessed. However, LS group demonstrated the highest average daily feed intake (ADFI), while CS group exhibited the lowest feed conversion ratio (FCR) throughout the experiment (Table 1).

### 3.2 Concentration of Faecal SCFAs

As shown in Table 2, the faecal concentration of acetate in LS group showed a tendency to be higher (*p* = 0.091) compared to CT group at 35 days. However, at 49 days, there were no differences in the concentrations of SCFAs.

**Table 2:** Concentration of SCFAs in faecal samples of weaned piglets at 35 and 49 days.

	Items, mmol	CT group	CS group	LS group	*p*‐value (CT‐CS)	*p*‐value (CT‐LS)	*p*‐value (CS‐LS)
35 days	Succinate	0.006 ± 0.002	0.003 ± 0.001	0.006 ± 0.001	0.251	0.957	0.375
	Lactate	0.051 ± 0.011	0.040 ± 0.010	0.094 ± 0.033	0.923	0.354	0.201
	Acetate	0.583 ± 0.047	0.722 ± 0.058	0.753 ± 0.053	0.185	0.091	0.910
	Propionate	0.234 ± 0.017	0.220 ± 0.023	0.250 ± 0.026	0.896	0.881	0.629
	Isobutyrate	0.020 ± 0.002	0.015 ± 0.002	0.015 ± 0.005	0.506	0.525	0.999
	Butyrate	0.123 ± 0.014	0.113 ± 0.017	0.121 ± 0.025	0.937	0.999	0.952
	Isovalerate	0.086 ± 0.011	0.047 ± 0.007	0.075 ± 0.023	0.200	0.854	0.432
	Valerate	0.076 ± 0.014	0.046 ± 0.010	0.066 ± 0.023	0.429	0.915	0.665
	Total	1.190 ± 0.069	1.211 ± 0.101	1.400 ± 0.104	0.986	0.276	0.346
49 days	Succinate	0.005 ± 0.002	0.006 ± 0.002	0.006 ± 0.001	0.868	0.971	0.959
	Lactate	0.019 ± 0.005	0.023 ± 0.005	0.041 ± 0.023	0.979	0.525	0.644
	Acetate	0.581 ± 0.088	0.684 ± 0.065	0.664 ± 0.024	0.513	0.642	0.975
	Propionate	0.280 ± 0.053	0.236 ± 0.024	0.270 ± 0.023	0.658	0.977	0.779
	Isobutyrate	0.025 ± 0.006	0.020 ± 0.006	0.023 ± 0.005	0.856	0.983	0.933
	Butyrate	0.137 ± 0.028	0.129 ± 0.027	0.136 ± 0.019	0.970	1.000	0.976
	Isovalerate	0.066 ± 0.015	0.043 ± 0.017	0.078 ± 0.017	0.586	0.875	0.320
	Valerate	0.069 ± 0.021	0.053 ± 0.018	0.053 ± 0.015	0.809	0.808	1.000
	Total	1.236 ± 0.222	1.205 ± 0.120	1.284 ± 0.072	0.989	0.972	0.929

*Note*: The mean and SEM are indicated. *n* = 6 per group.

Abbreviations: CS group, weaned piglets supplemented with early‐stage diet added 30 ppm colistin sulphate; CT group, weaned piglets supplemented with early‐stage diet; LS group, weaned piglets supplemented with early‐stage diet added heat‐treated *Lactobacillus sakei* HS‐1 strain at a rate of 2.0 × 10^5^ cells/g.

### 3.3 Concentration of Plasma Amino Acids

The plasma amino acid levels at 35 and 49 days are shown in Tables 3 and 4. Serine and histidine were significantly higher (*p* < 0.05) at 35 days in LS group compared to CT group. Alanine, cysteine and methionine also showed a tendency to be higher (*p* < 0.1) at 35 days in LS group compared to CT group. Histidine showed a tendency to be higher (*p* = 0.054) at 35 days in LS group compared to CS group. Proline showed a tendency to be higher (*p* = 0.092) at 49 days in LS group than CT group, and was significantly higher (*p* = 0.011) at 49 days in LS group than CS group. Lysine showed a tendency to be higher (*p* = 0.072) at 49 days in LS group than CS group.

**Table 3:** Concentration of plasma amino acids of weaned piglets at 35 days.

Items, mM	CT group	CS group	LS group	*p*‐value (CT‐CS)	*p*‐value (CT‐LS*)*	*p*‐value (CS‐LS)
Asp	0.013 ± 0.001	0.015 ± 0.002	0.012 ± 0.002	0.636	0.994	0.571
Glu	0.073 ± 0.009	0.092 ± 0.018	0.078 ± 0.011	0.565	0.955	0.739
Ser	0.060 ± 0.006	0.094 ± 0.017	0.123 ± 0.019	0.268	0.025	0.392
Gly	0.332 ± 0.043	0.472 ± 0.035	0.446 ± 0.064	0.138	0.257	0.921
His	0.022 ± 0.002	0.027 ± 0.002	0.035 ± 0.002	0.249	0.002	0.054
Thr	0.085 ± 0.010	0.090 ± 0.007	0.109 ± 0.017	0.951	0.353	0.516
Ala	0.280 ± 0.020	0.354 ± 0.029	0.381 ± 0.033	0.180	0.052	0.768
Pro	0.054 ± 0.004	0.041 ± 0.006	0.057 ± 0.009	0.923	0.379	0.218
Arg	0.128 ± 0.011	0.163 ± 0.012	0.177 ± 0.024	0.325	0.130	0.831
Val	0.111 ± 0.013	0.141 ± 0.010	0.146 ± 0.021	0.401	0.287	0.968
Cys	0.006 ± 0.001	0.007 ± 0.001	0.009 ± 0.001	0.721	0.098	0.341
Met	0.028 ± 0.002	0.034 ± 0.003	0.042 ± 0.006	0.629	0.061	0.292
leu	0.069 ± 0.007	0.073 ± 0.005	0.084 ± 0.009	0.908	0.326	0.549
Ile	0.088 ± 0.013	0.111 ± 0.009	0.114 ± 0.019	0.484	0.410	0.990
Phe	0.057 ± 0.004	0.060 ± 0.004	0.063 ± 0.007	0.887	0.719	0.947
Lys	0.093 ± 0.012	0.085 ± 0.011	0.090 ± 0.018	0.915	0.986	0.968
Tyl	0.049 ± 0.006	0.057 ± 0.005	0.062 ± 0.010	0.680	0.399	0.879
Total	1.548 ± 0.139	1.918 ± 0.118	2.029 ± 0.248	0.330	0.168	0.899

*Note:* The mean and SEM are indicated. *n* = 6 per group.

Abbreviations: CS group, weaned piglets supplemented with early‐stage diet added 30 ppm colistin sulphate; CT group, weaned piglets supplemented with early‐stage diet; LS group, weaned piglets supplemented with early‐stage diet added heat‐treated *Lactobacillus sakei* HS‐1 strain at a rate of 2.0 × 10^5^ cells/g.

**Table 4:** Concentration of plasma amino acids of weaned piglets at 49 days.

Items, mM	CT group	CS group	LS group	*p*‐value (CT‐CS)	*p*‐value (CT‐LS*)*	*p*‐value (CS‐LS)
Asp	0.020 ± 0.003	0.023 ± 0.003	0.027 ± 0.005	0.824	0.458	0.807
Glu	0.114 ± 0.019	0.133 ± 0.019	0.139 ± 0.020	0.760	0.628	0.973
Ser	0.083 ± 0.011	0.083 ± 0.009	0.106 ± 0.005	1.000	0.155	0.161
Gly	0.410 ± 0.047	0.403 ± 0.035	0.494 ± 0.025	0.991	0.273	0.224
His	0.032 ± 0.005	0.034 ± 0.003	0.043 ± 0.004	0.973	0.175	0.249
Thr	0.145 ± 0.022	0.148 ± 0.020	0.184 ± 0.015	0.995	0.340	0.386
Ala	0.341 ± 0.038	0.345 ± 0.043	0.429 ± 0.069	0.999	0.469	0.498
Pro	0.073 ± 0.012	0.060 ± 0.005	0.100 ± 0.007	0.549	0.092	0.011
Arg	0.185 ± 0.021	0.185 ± 0.017	0.235 ± 0.028	1.000	0.272	0.271
Val	0.169 ± 0.019	0.166 ± 0.016	0.201 ± 0.014	0.992	0.384	0.325
Cys	0.010 ± 0.001	0.011 ± 0.001	0.009 ± 0.003	0.935	0.951	0.794
Met	0.034 ± 0.006	0.036 ± 0.003	0.048 ± 0.005	0.965	0.126	0.192
leu	0.098 ± 0.012	0.087 ± 0.007	0.114 ± 0.008	0.688	0.470	0.138
Ile	0.138 ± 0.017	0.135 ± 0.011	0.160 ± 0.011	0.983	0.503	0.405
Phe	0.071 ± 0.007	0.074 ± 0.003	0.084 ± 0.005	0.950	0.269	0.410
Lys	0.134 ± 0.021	0.117 ± 0.012	0.170 ± 0.012	0.715	0.272	0.072
Tyl	0.073 ± 0.008	0.072 ± 0.006	0.091 ± 0.007	0.986	0.215	0.167
Total	2.131 ± 0.222	2.111 ± 0.156	2.634 ± 0.156	0.997	0.155	0.135

*Note:* The mean and SEM are indicated. *n* = 6 per group.

Abbreviations: CS group, weaned piglets supplemented with early‐stage diet added 30 ppm colistin sulphate; CT group, weaned piglets supplemented with early‐stage diet; LS group, weaned piglets supplemented with early‐stage diet added heat‐treated *Lactobacillus sakei* HS‐1 strain at a rate of 2.0 × 10^5^ cells/g.

### 3.4 Correlation Analysis of ADG, Total Plasma Amino Acids, and Faecal Acetate

Spearman correlation analysis was further conducted to investigate whether faecal SCFAs and plasma amino acids are linked to growth performance. As shown in Supporting Information S1: Figure S1A,B, faecal acetate concentration (*r* = 0.54, *p* = 0.01) and total plasma amino acids (*r* = 0.74, *p* < 0.01) had a significant correlation with ADG throughout the experimental period.

### 3.5 Analysis of Immune Function With ELISA

**Figure 2:**
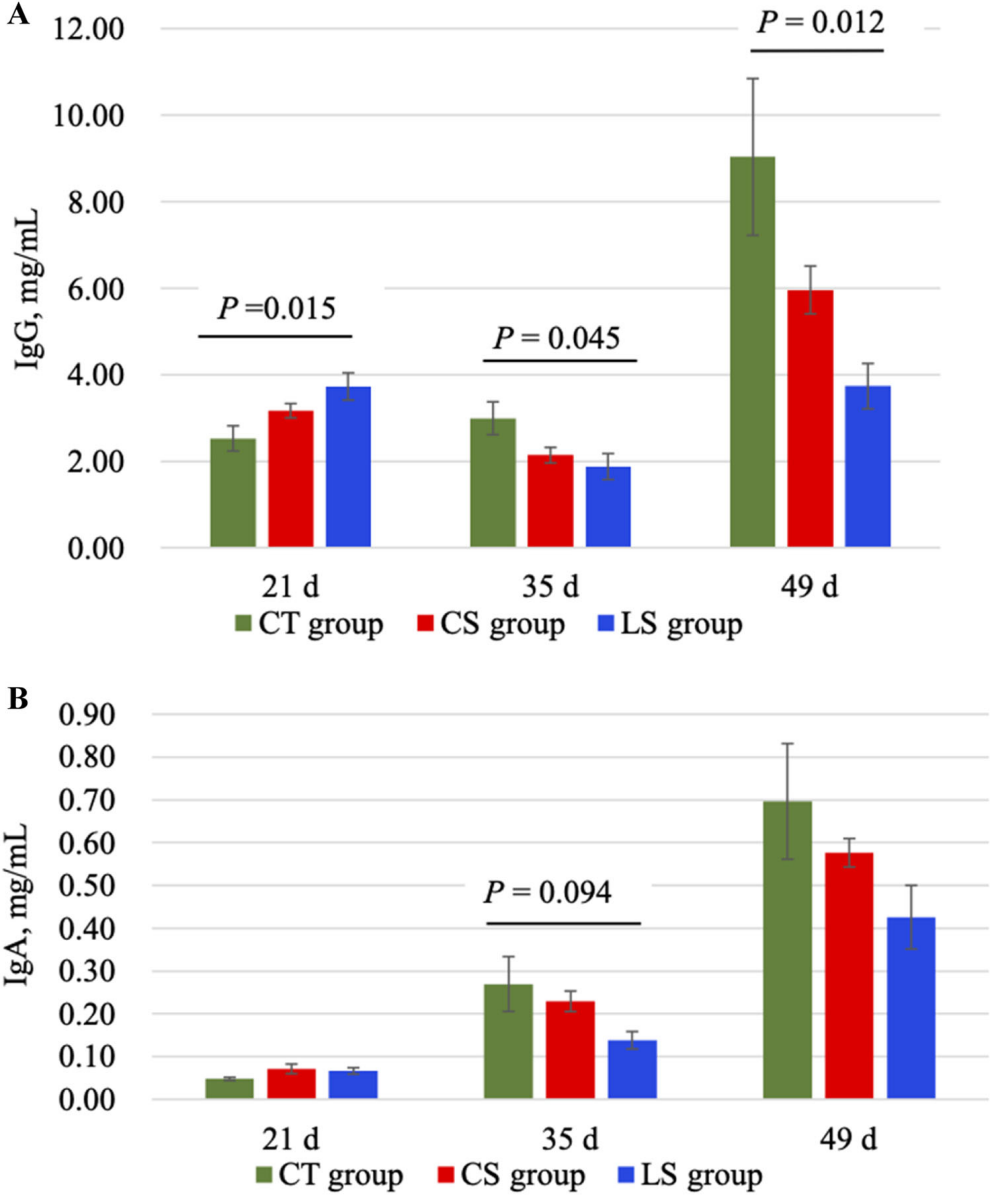
Concentrations of plasma (A) IgG and (B) IgA of weaned piglets at 21, 35, and 49 days. The mean and SEM are shown. [Color figure can be viewed at wileyonlinelibrary.com]

Concentrations of IgG and IgA in plasma are shown in Figure 2A,B. Plasma IgG concentrations of LS group at 35 and 49 days were significantly lower than CT group (*p* = 0.045 and *p* = 0.012, respectively). The plasma IgA concentration of LS group at 35 days showed a tendency to be lower than CT group (*p* = 0.094). However, there were no significant differences in the sIgA concentrations in intestinal mucosa (Supporting Information S1: Figure S2).

### 3.6 Comparison of Faecal Microbiota Composition and Diversity

**Figure 3:**
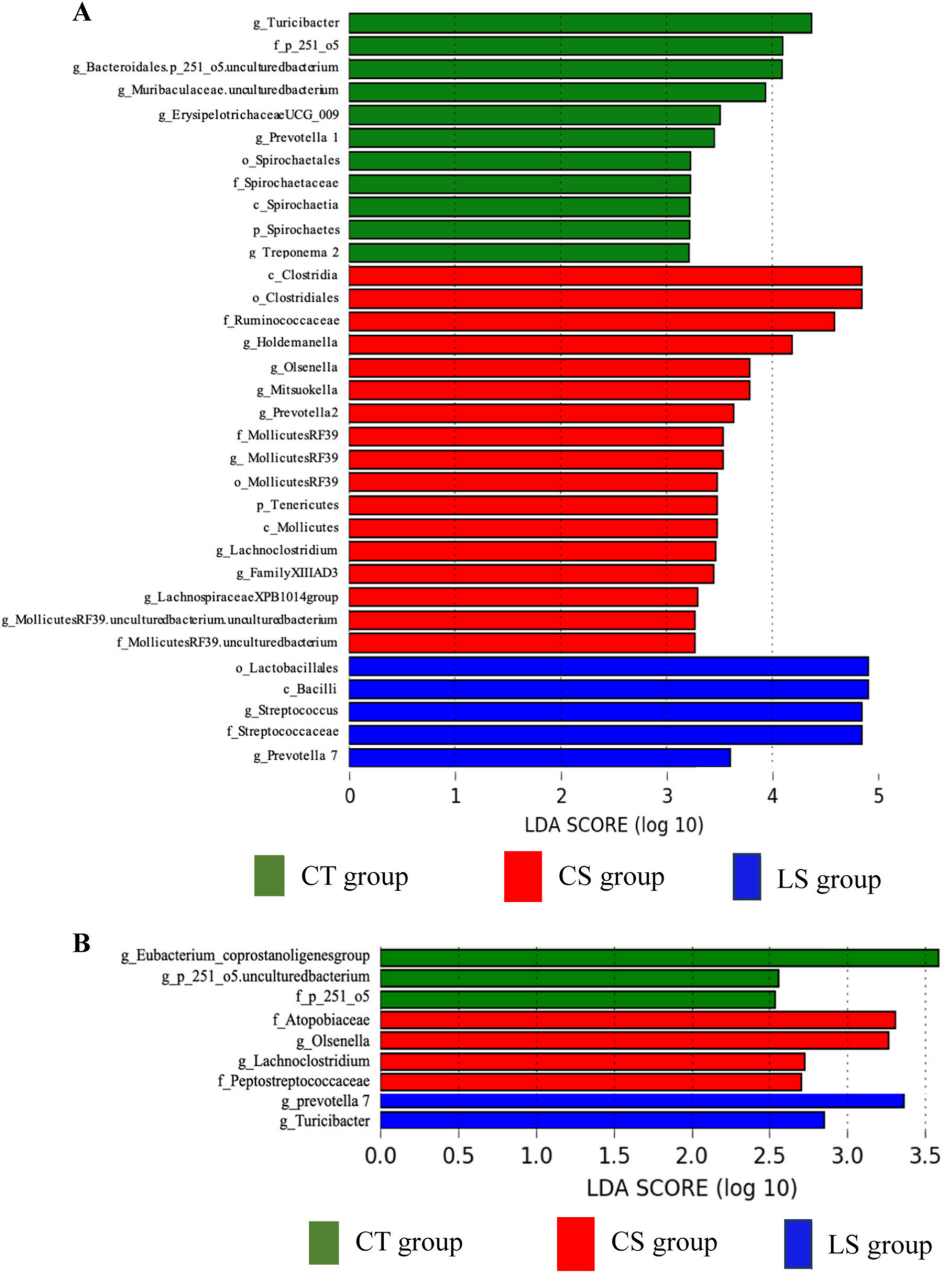
LEfSe method identified the most differentially abundant taxa enriched in the faecal microbiota of the groups. Histogram of the taxa with significant differences that have an LDA score (log_10_) ≥ 2.0 calculated for features differentially abundant in the groups. (A) Differences in taxa at 35 days. (B) Differences in taxa at 49 days. [Color figure can be viewed at wileyonlinelibrary.com]

LEfSe analysis was performed to identify biomarkers that distinguish between the groups at 35 and 49 days. As shown in Figure 3A, at 35 days, 33 bacterial taxa were identified in each of the three groups with significantly different taxonomic levels compared to the other groups. g_Streptococcus, and g_Prevotella 7, were significantly enriched in LS group. g_Holdemanella, g_Olsenella, g_Mitsuokella, g_Prevotella 2, g_Mollicutes RF39, g_Lachnoclostridium, Family Ⅷ AD3, g_MollicutesRF39_uncultured bacterium_uncultured bacterium, and g_Lachnospiraceae_XPB1014group were enriched in CS group, and g_Turicibacter, g_Bacteroidales.p_251_o5_uncultured bacterium, g_ErysipelotrichaceaeUCG_009, g_Prevotella 1, and g_Treponema 2 were significantly higher in CT group. As shown in Figure 3B, at 49 days, there were nine bacterial taxa distinguishing the groups. g_Prevotella 7 and g_Turicibacter were significantly enriched in LS group and g_Olsenella and g_Lachnoclostridium were enriched in CS group. g_Eubacterium_coprostanoligenes group and g_p_251_o5_uncultured bacterium were significantly higher in CT group. Supporting Information S1: Figures S3 and S4 show microbial composition at phylum and genus level for phyla present at an abundance > 0.1% in at least one group and compared for different time points. Observed_features of LS group at 35 days was significantly lower than CS group (*p* = 0.029) (Supporting Information S1: Figure S5A), and Shannon's index of LS group at 35 days was significantly lower than CT group and CS group (*p* = 0.015, *p* < 0.01, respectively) (Supporting Information S1: Figure S5B). Pielou_evenness of LS group at 35 days was significantly lower than CS group (*p* < 0.01) and tended to be lower than CT group (*p* = 0.056). Pielou_evenness of CS group at 35 days tended to be higher than CT group (*p* = 0.084) (Supporting Information S1: Figure S5C). On the other hand, the indices in the three groups did not differ at 49 days (Supporting Information S1: Figure S6A–C). Permutational multivariate analysis of variance in the three groups based on weighted UniFrac distance revealed that there were no significant dissimilarities among the three groups at either 35 or 49 days (Supporting Information S1: Figure S7).

### 3.7 Functional Pathway Predictions Using PICRUSTs2

**Figure 4:**
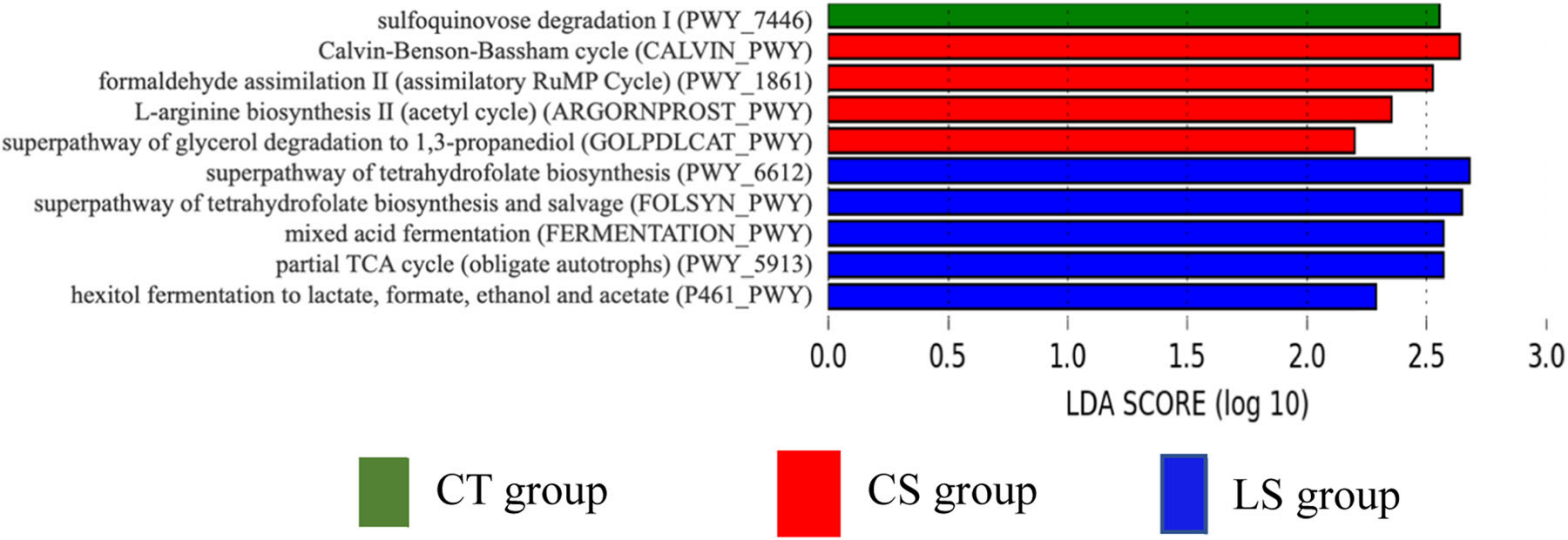
The metabolic pathways with linear discriminant analysis (LDA) score ≥ 2.0 using the MetaCyc database between the groups. [Color figure can be viewed at wileyonlinelibrary.com]

PICRUSTs2 was used to predict metagenomic functions in the microbial community from amplicon sequencing data obtained at 35 and 49 days. The metabolic pathways with linear discriminant analysis (LDA) score ≥ 2.0, as based on the MetaCyc database, are shown in Figure 4. Consequently, a total of 10 pathways were identified as significantly different between groups. Pathways of folate biosynthesis, mixed acid fermentation, TCA cycle, and sugar alcohols degradation were upregulated in LS group compared with the other groups. The pathways of Calvin‐Benson‐Bassham cycle, formaldehyde assimilation, L‐arginine degradation, and glycerol degradation were enriched in CS group compared to the other groups. Sulfoquinovose degradation was higher in CT group, and relatively downregulated in the two treatment groups.

### 3.8 Correlation Between Plasma Amino Acids or Faecal SCFAs and Gut Microbiota

**Figure 5:**
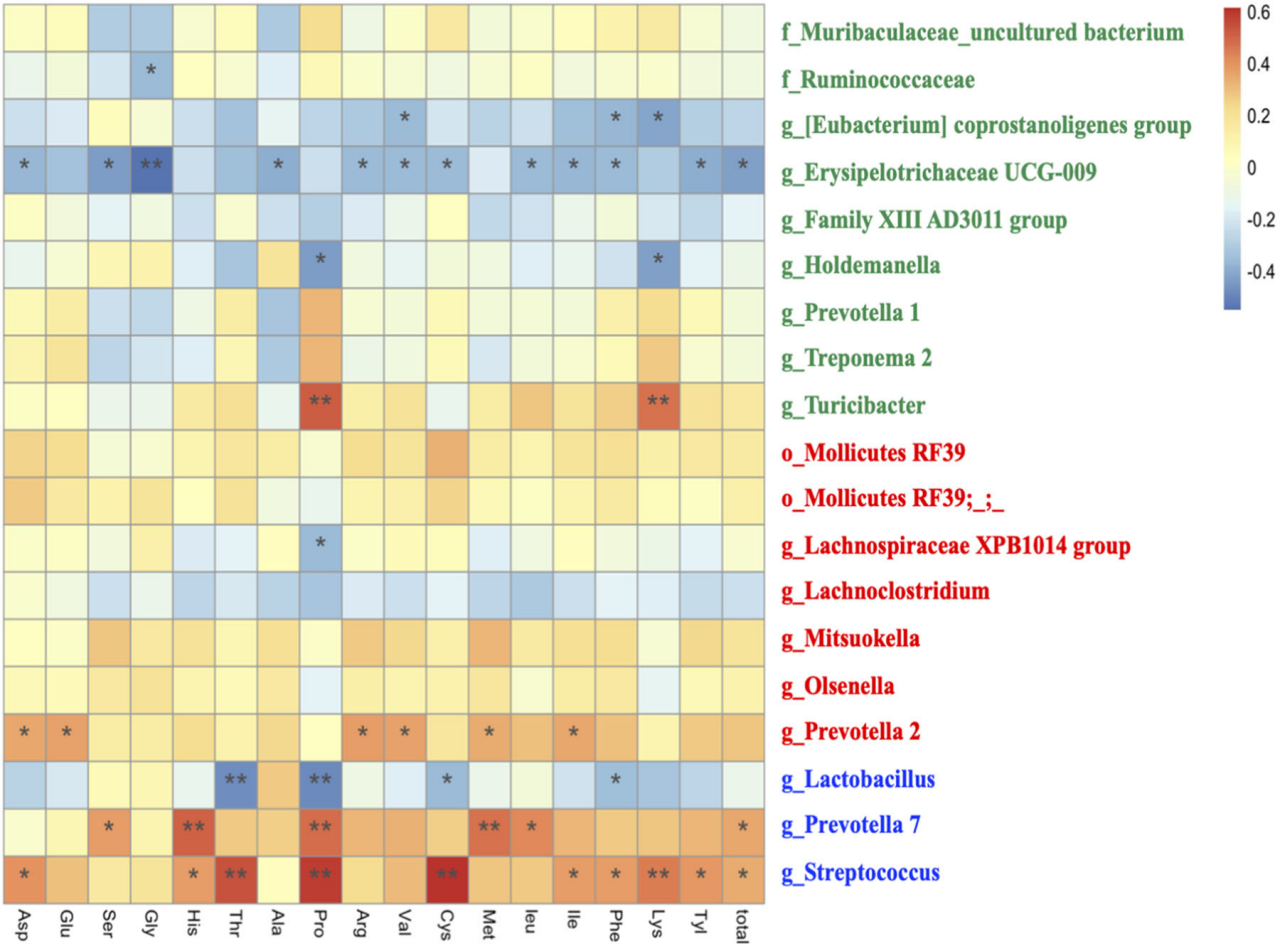
Heatmap of the correlation between major microbiota changed in the groups and plasma amino acids. Spearman's test was used to calculate the correlation coefficient. The negative correlation was expressed by blue colour, and the positive correlation was expressed by red colour. Green characters indicate microbiota with significantly higher abundance in the CT group, red characters in the CS group, and blue characters in the LS group. ** means *p* < 0.01. * means *p* < 0.05. [Color figure can be viewed at wileyonlinelibrary.com]

**Figure 6:**
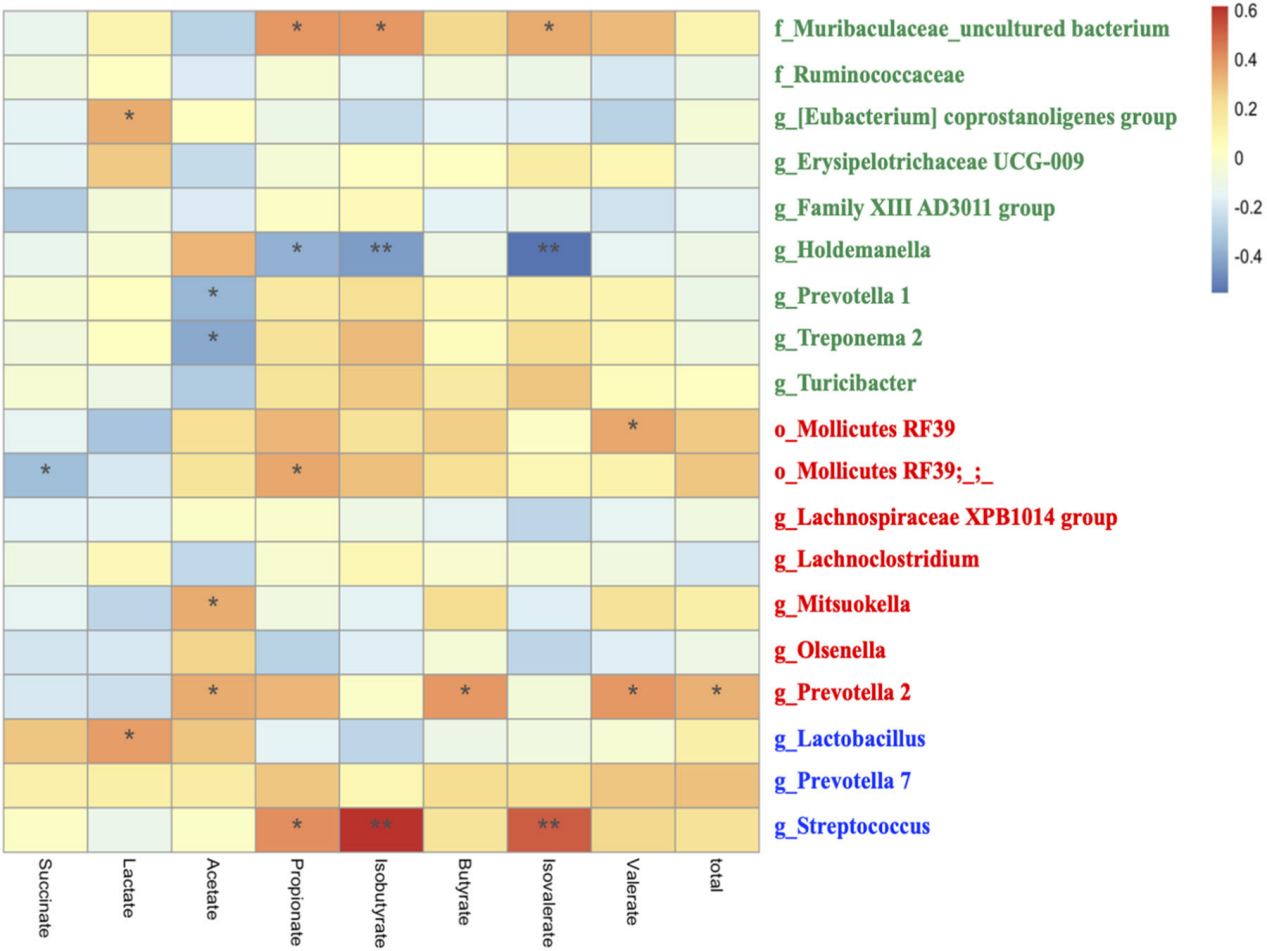
Heatmap of the correlation between major microbiota changed in the groups and faecal short‐chain fatty acids. Spearman's test was used to calculate the correlation coefficient. The negative correlation was expressed by blue colour, and the positive correlation was expressed by red colour. Green characters indicate microbiota with significantly higher abundance in the CT group, red characters in the CS group, and blue characters in the LS group. ** means *p* < 0.01. * means *p* < 0.05. [Color figure can be viewed at wileyonlinelibrary.com]

Finally, Spearman correlation analysis was conducted to presume the relationships between bacteria and plasma amino acids or faecal SCFAs (Figures 5 and 6). As for plasma amino acids, g_Streptococcus was positively correlated to Thr, Pro, Cys and Lys (*p* < 0.01). g_Prevotella 7 was positively correlated to His, Pro, Met (*p* < 0.01). g_Turicibacter was positively correlated to Pro and Lys (*p* < 0.01). g_Lactobacillus was negatively correlated to Thr and Pro (*p* < 0.01). g_Erysipelotrichaceae UCG‐009 was negatively correlated to Gly (*p* < 0.01) (Figure 5). Concerning SCFAs, g_Lactobacillus was positively correlated to lactate (*p* < 0.05). g_Steptococcus was positively correlated to propionate (*p* < 0.05), isobutyrate (*p* < 0.01) and isovalerate (*p* < 0.01), while g_Holdemanella was negatively correlated to propionate (*p* < 0.05), isobutyrate (*p* < 0.01) and isovalerate (*p* < 0.01) (Figure 6).

## 4 Discussion

### 4.1 Potential of HT‐LS as a Growth Promoter

In this study, we used crossbred weaned piglets to evaluate the potential of HT‐LS as a growth promoter specifically comparing it to commonly used AGP, CS, in swine production. The research aimed to evaluate the impact of HT‐LS on the growth performance of weaned piglets as well as its influence on gut microbial composition and nutritional aspects. Several reports have shown that feeding inactivated probiotics modulates immune function and improves growth performance in weaned piglets. For example, the supplementation of heat‐sterilized *Lactobacillus rhamnosus* at a rate of 1 × 10^6^ cells/g of feed led to improved apparent total tract digestibility, elevated ADG, and an enhanced gain to feed ratio (Kang et al. 2021). Similarly, feeding heat‐sterilized *Enterococcus faecium* strain NHRD IHARA at a rate of 2 × 10^10^ cells/kg of feed resulted in weaned piglets significantly improved growth performance and improved villus growth in the jejunum (Sukegawa et al. 2014). However, there were no previous reports of feeding *Lactobacillus sakei* to weaned piglets. In this study, LS group showed a significant ADG improvement compared to CT group throughout the experimental period, and even showed a tendency for better performance compared to CS group in the period 42–49 days. Thus, HT‐LS could be utilized as a growth promoter at postweaning period.

### 4.2 HT‐LS Could Improve Nutritional Status

Furthermore, LS group showed a tendency to be higher faecal acetate concentrations compared CT group. SCFAs are major microbial metabolites derived from carbohydrates in the gut (Duarte and Kim 2022), and contribute significantly to energy, especially in pigs, where their energy contribution is estimated to range from 30% to 76% (Nakatani et al. 2018). In previous reports, the faecal SCFA concentrations correlated with digestive tract development and postweaning weight gain (Zhou et al. 2019). Therefore, the increased abundance of acetate in the gut may have contributed to growth performance improvement in the present study.

Amino acids are fundamental building blocks of protein in animals and play a crucial role in promoting growth. Recent reviews have underscored the significance of dietary amino acids for gut health and growth performance in weaned piglets (Liao 2021). Feeding amino acids to piglets has been linked to improved health and growth (Lin et al. 2014). It has been reported that pigs affected by pathogenic bacteria reduced growth performance and significantly lower blood amino acid concentrations, but the supplementation of functional amino acids rescues growth under such conditions (Trevisi et al. 2009; Rodrigues et al. 2021). In this study, several amino acids were significantly higher or tended to be higher in LS group than in the other groups. These results suggest that HT‐LS feeding potentially enhanced amino acid absorption from the intestine and/or facilitated amino acid production by gut microbiota. Although, as shown in Figure 4, functional pathway prediction of microbiota could not reveal the reason for the increase in individual amino acids in the LS group, the effects of feeding live microorganisms or probiotics on amino acid metabolism, particularly the impact on *Clostridium butyricum* (Liang et al. 2021), has been reported. Our study stands as one of the first to suggest that heat‐treated probiotics may modulate amino acid metabolism in weaned piglets, albeit necessitating further mechanistic investigation.

### 4.3 HT‐LS Could Affect Immune Function

In pig production, the weaning stage is an important period as piglets transition from passive to adaptive immunity. Blood immunoglobulin concentrations peak immediately after birth, gradually decline toward weaning, and then increase again due to active immunity (Bourne 1973; Frenyó et al. 1981). Thus, weaned piglets become susceptible to intestinal infections from various pathogens. Feeding heat‐treated *E. faecium* NHRD IHARA strain has been shown to increase IgA concentrations in blood and faeces (Sukegawa et al. 2014). Similarly, a study with mice has reported that HT‐LS increased secretory IgA concentrations in the gut, enhanced the expression of anti‐inflammatory cytokines such as IL‐10, and showed local antimicrobial activity against pathogenic bacteria, including *Escherichia coli* (Ghoneum and Abdulmalek 2021). On the other hand, the present study demonstrated that plasma IgG levels in LS group at 35 and 49 days were significantly lower than CT group, with IgA levels at 35 days also displaying a tendency to decrease. The plasma levels of immunoglobulin in the piglets in this study were not significantly higher than in previous reports (Tuchscherer et al. 2012; Augustyniak, Czyżewska‐Dors, and Pomorska‐Mól 2024), and the piglets did not show any clinical signs during the experimental period, suggesting that the piglets were raised in an appropriate environment and under normal condition. However, the effects on plasma immunoglobulin in this study differed from the previous reports. Intestinal infections are often subclinical, but chronic inflammation can negatively impact growth performance and overall farm management (Alarcon, Rushton, and Wieland 2013). Chronic inflammation suppresses appetite, consumes nutrients to maintain the immune system, and increases nutrient requirements for growth (Broom and Kogut 2018). HT‐LS may stimulate the local immune response in the gut, thereby suppressing the chronic inflammatory response caused by infections and allowing nutrients to be used for growth. It is also known that SCFAs, whose production was enhanced by HT‐LS feeding, have bioactive functions such as intestinal antimicrobial activity (Lauridsen 2020) and reduction of ileal inflammatory cytokines (Xu et al. 2016) in neonatal piglets. Acetate, for instance, prevented *E. coli* from invading the colonic mucosa (Takeuchi et al. 2021), and lowered pH in the faeces, thereby diminishing *E. coli* populations (Corrier et al. 1990).

### 4.4 Changes in Gut Microbiota Composition and Effects on Nutrient Metabolism

Additionally, 16S rRNA gene amplicon sequencing was conducted to analyse the faecal gut microbiota composition at 35 and 49 days. The results showed a higher relative abundance of lactic acid bacteria like o_Lactobacillales and g_Streptococcus in LS group compared to the other groups at 35 days. Previous reports have shown that feeding heat‐treated *Lactobacillus* sp. to pigs increased the abundance of lactic acid bacteria in the gut (Ryu et al. 2022; Xu, Duarte, and Kim 2022). Although the mechanism of those increases is unclear, we observed similar increase in the bacterial groups including lactic acid bacteria and assumed that feeding HT‐LS might have caused a change in the gut microbiota. LS group also displayed a significantly higher representation of the genus g_Prevotella 7 at both 35 and 49 days. The genus is known for metabolizing nonstarch plant‐derived polysaccharides into SCFAs (Ivarsson et al. 2014) and has been associated with increased feed intake in pigs (Yang et al. 2018). Interestingly, a significant positive correlation was observed between g_Streptococcus and propionate, isobutyrate, and isovalerate. *Streptococcus* spp. are regarded as components of a healthy gut flora and are linked to piglet weight (Li et al. 2020; Rhouma et al. 2021). Research has suggested that SCFA levels rise in tandem with *Streptococcus* genus abundance (Sun et al. 2022), which could provide a mechanistic explanation for the growth performance improvement observed in the study. Metagenomic analysis of microbiota predicted by PICRUSt2 also revealed enriched metabolic pathways in LS group related to mixed acid fermentation, partial TCA cycle (obligate autotrophs), and hexitol fermentation to lactate, formate, ethanol, and acetate, suggesting activated microbial production of SCFAs.

We also found correlations between changes in major microbiota composition and plasma amino acids or faecal SCFA levels. g_Streptococcus and g_Prevotella 7, which increased in LS group, showed a positive correlation. Gut microbiota probably alter the bioavailability of amino acids by utilizing certain amino acids derived from the gastrointestinal tract and endogenous proteins, thus supplying amino acids to the host (Neis, Dejong, and Rensen 2015; Lin et al. 2017). Although the detailed role of microbiota in host amino acid metabolism is not clear, *Clostridium* clusters, the *Bacillus*‐*Lactobacillus*‐*Streptococcus* group in the small intestine of humans and pigs, has been reported to be the main species involved in amino acid fermentation (Dai 2011). *Prevotella copri* has been associated with increased serum concentrations of branched‐chain amino acids and aromatic amino acids in pigs (Chen et al. 2021). In addition, the administration of *Lactobacillus plantarum* JDFM LP11 strain regulated the gut microbiota in weaned piglets, activating branched‐chain amino acids synthesis and butyrate metabolism (Shin et al. 2019). The present study suggests that heat‐treated probiotics could potentially influence amino acid metabolism via gut microbiota modulation. Conversely, certain bacterial groups, which were relatively abundant in CT group, displayed negative correlations with growth performance and nutritional aspects in weaned piglets. g_Prevotella 1 and g_Treponema 2 showed significant negative correlations with faecal acetate concentration. Prior study has indicated that piglets with elevated intestinal levels of *Treponema* 2 and *Spirochetes* had lower caecal acetic acid levels (Wang et al. 2019). *Spirochetes* include some important pathogenic bacteria for humans and pigs, such as leptospirosis and swine dysentery. Potential pathogens could affect SCFA levels in the gut and negatively impact growth performance in weaned piglets. On the other hand, a limitation of this study is that gut microbiota analysis was not performed at the beginning of the experiment (21 days). Although the pigs were randomly assigned to the groups among the piglets from the same sow and the cage effects can also occur even during the experiment, considering the cage effects, it is desirable to conduct gut microbiota analysis at baseline.

## 5 Conclusion

In the present study, the effectiveness of HT‐LS was compared and investigated with colistin, which is widely used as an AGP in weaned piglets. The results showed that feeding HT‐LS improved growth performance of weaned piglets compared to the control, and was comparable to an AGP. Our findings suggest that modifications in the gut microbiota, particularly an increase in lactic acid bacteria such as g_Streptococcus and g_Lactobacillus, contributed to the increase in intestinal SCFAs concentrations, and possibly the increase in plasma amino acids. Additionally, suppression of immunoglobulin production may also have contributed to improved growth performance. Feeding HT‐LS to weaned piglets could be a new strategy to replace AGPs.

## Ethics Statement

The authors confirm that the ethical policies of the journal, as noted on the journal's author guidelines page, have been adhered to and the appropriate ethical review committee approval has been received. The experiment was approved by the Animal Care and Use Committee of the University of Tokyo (P21‐031). The authors confirm that they have followed EU standards for the protection of animals used for scientific purposes [and feed legislation, if appropriate].

## Conflicts of Interest

HT‐LS used in this study was provided by Daiwa Pharmaceutical Co. Ltd. R Ninomiya is an employee of Daiwa Pharmaceutical Co. Ltd.

## Supporting information

Supporting information.

## Data Availability

The data that support the findings of this study are available from the corresponding author upon reasonable request.
