# Supplementary material for: Beneficial Role of Heat‐Treated Lactobacillus sakei HS‐1 on Growth Performance, Nutritional Status and Gut Microbiota in Weaned Piglets
Source: J Anim Physiol Anim Nutr (Berl). 2024 Oct 16;109(2):362–75. doi: 10.1111/jpn.14056 (PMC11919806; doi:10.1111/jpn.14056)
Supplement: Supplementary file 1 — Supporting information. [file JPN-109-362-s001.docx]

**Supplemented Figure 1A**

**
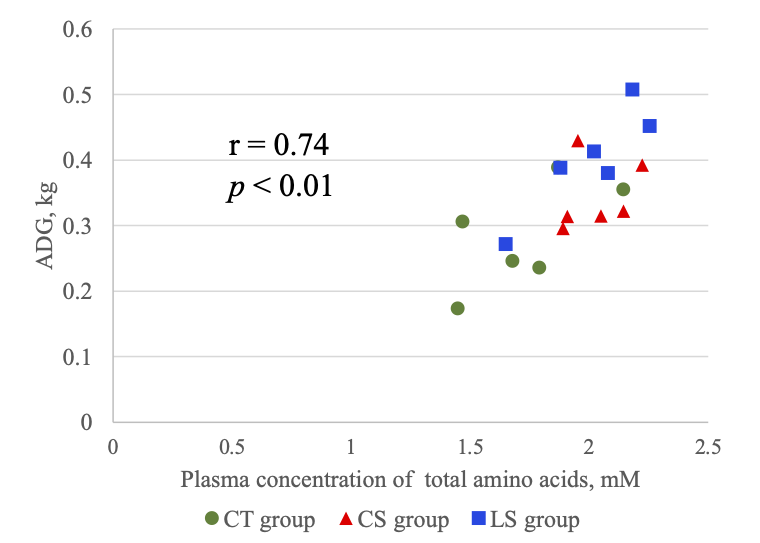
**

**Supplemented Figure 1B**

**
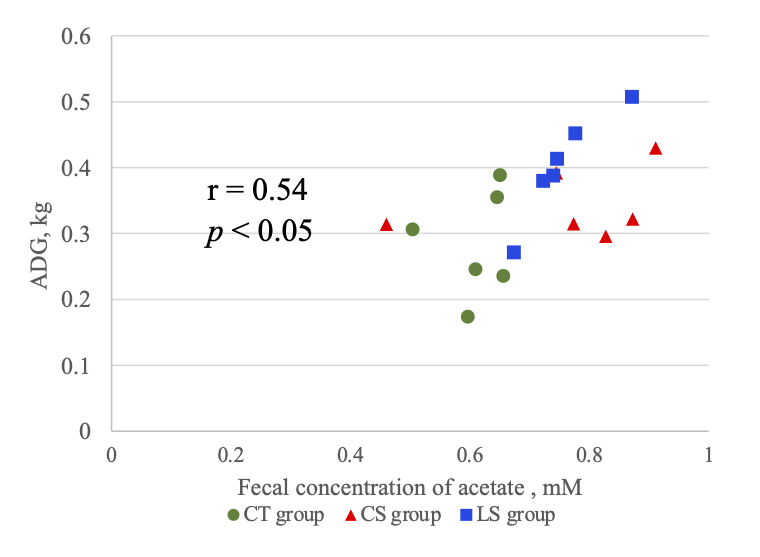
**

**Supplemented Figure 2**

**
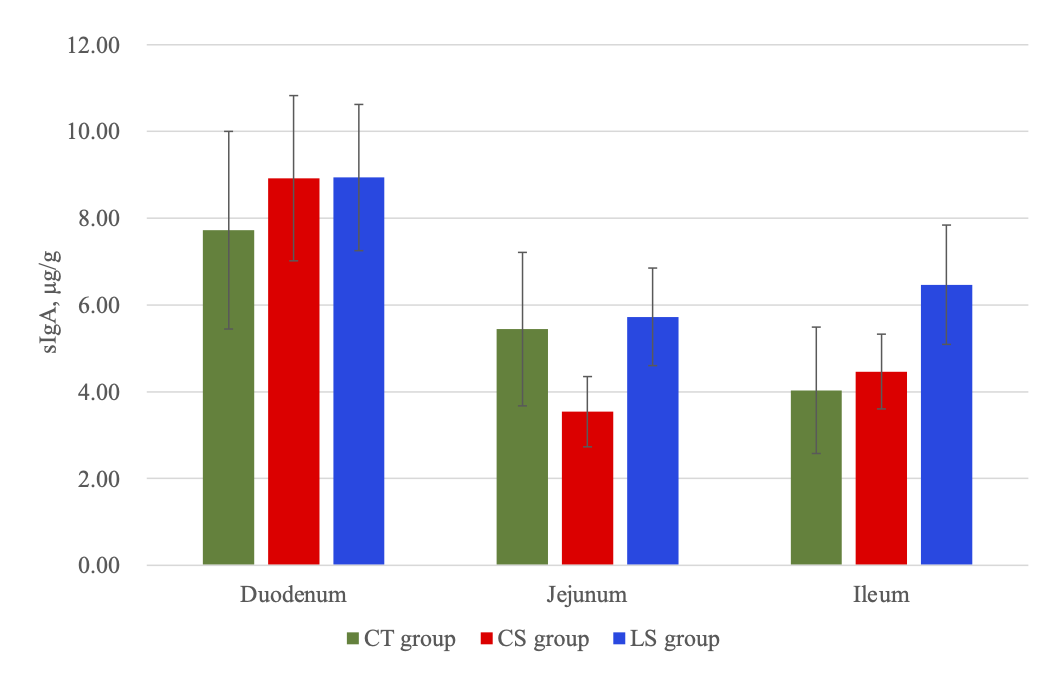
**

**Supplemented Figure 3A**

**
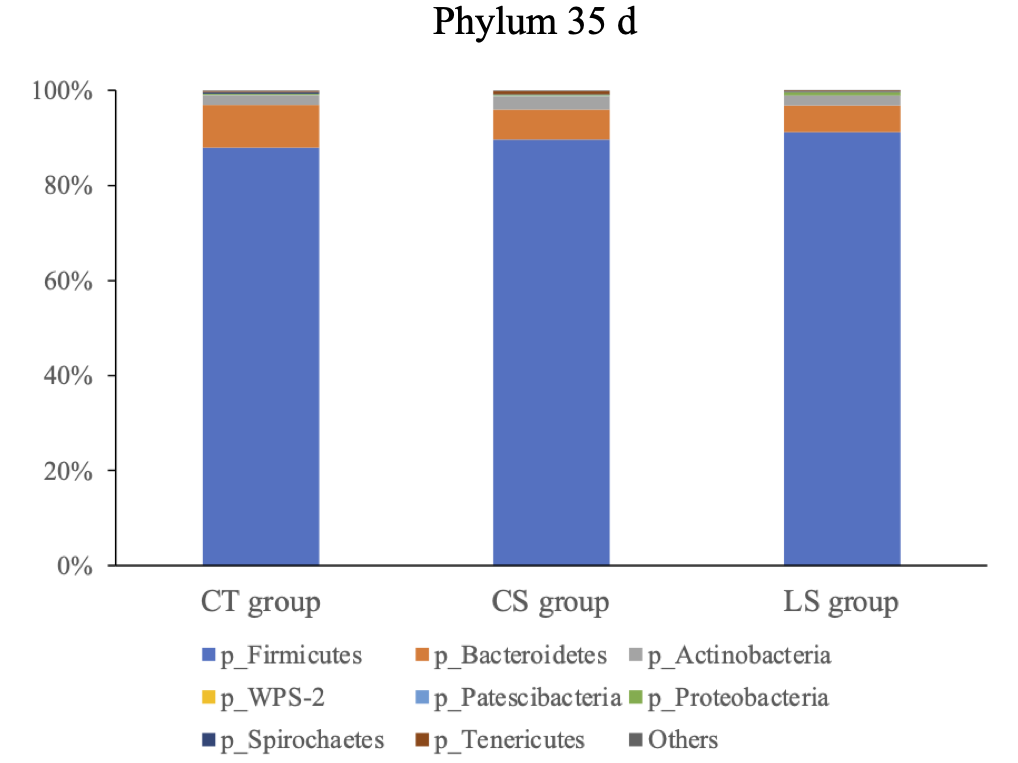
**

**Supplemented Figure 3B**

**
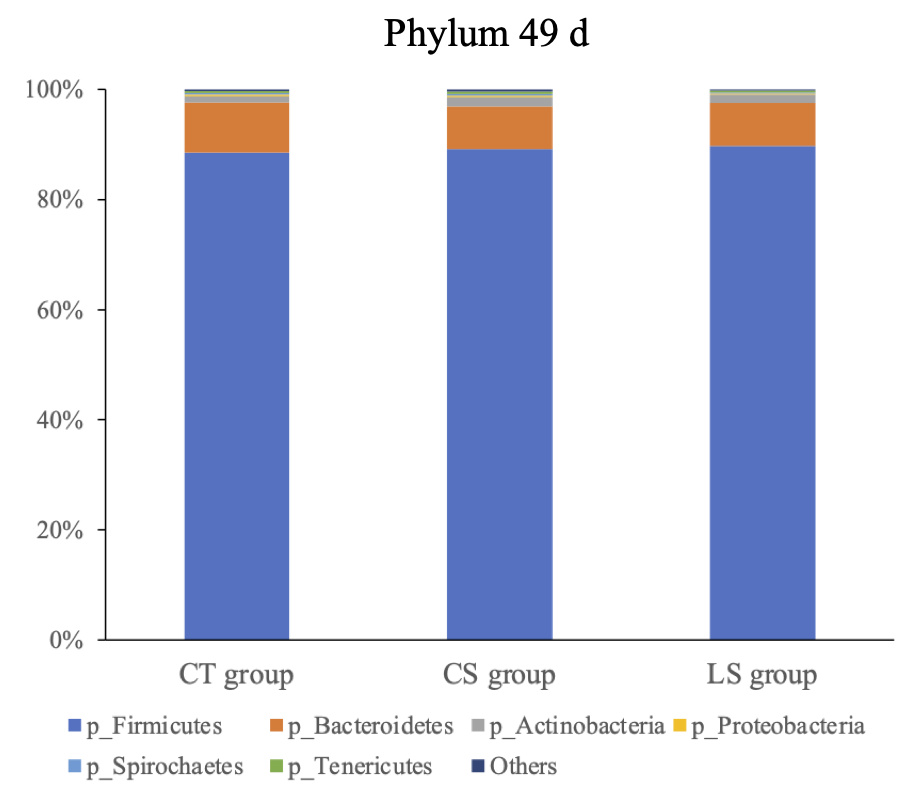
**

**Supplemented Figure 4A**

**
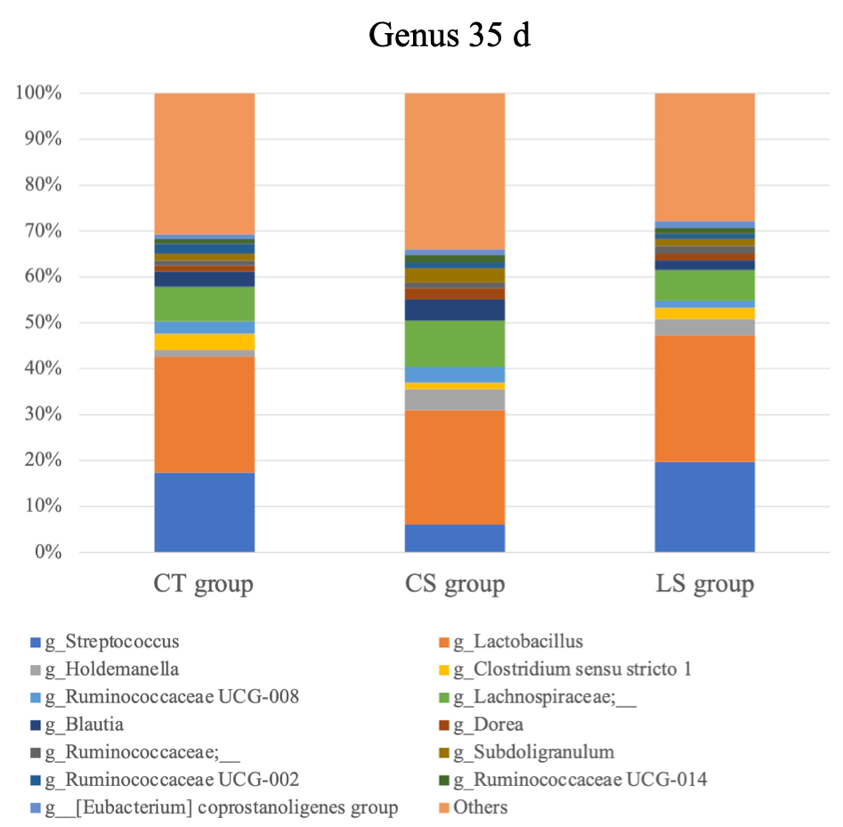
**

**Supplemented Figure 4B**

**
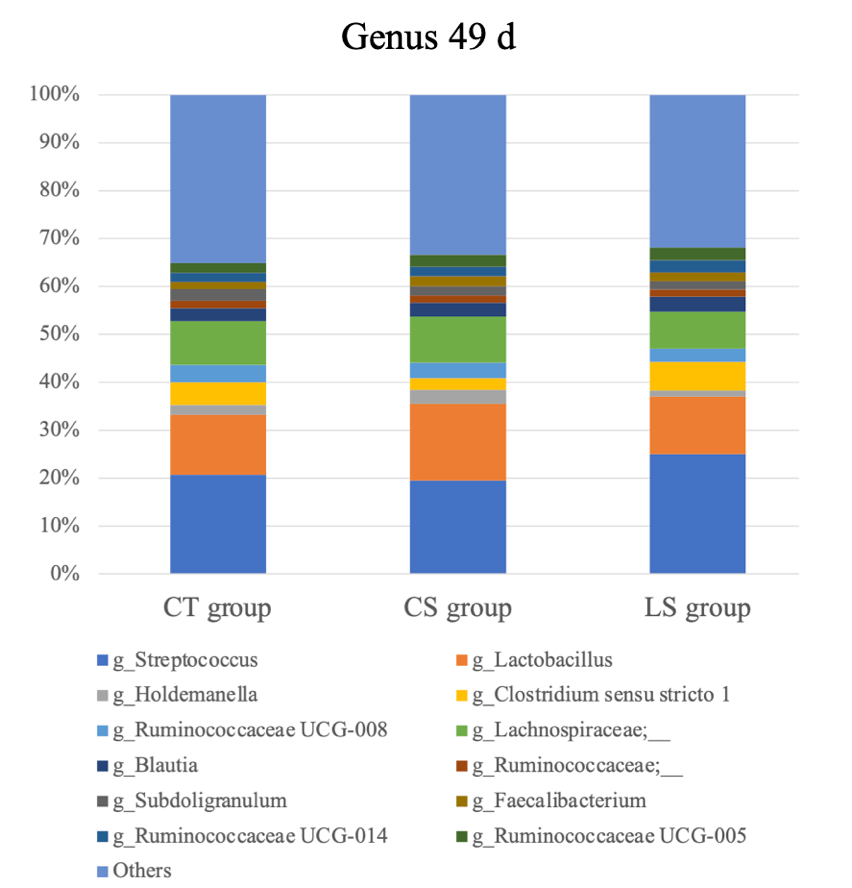
**

**Supplemented Figure 5A**

**
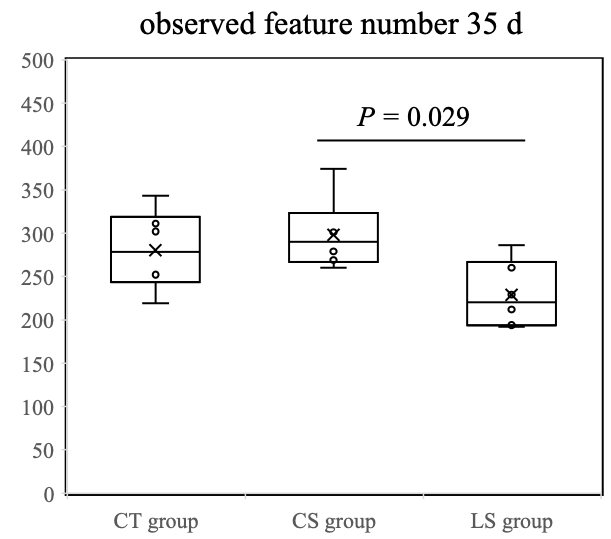
**

**Supplemented Figure 5B**

**
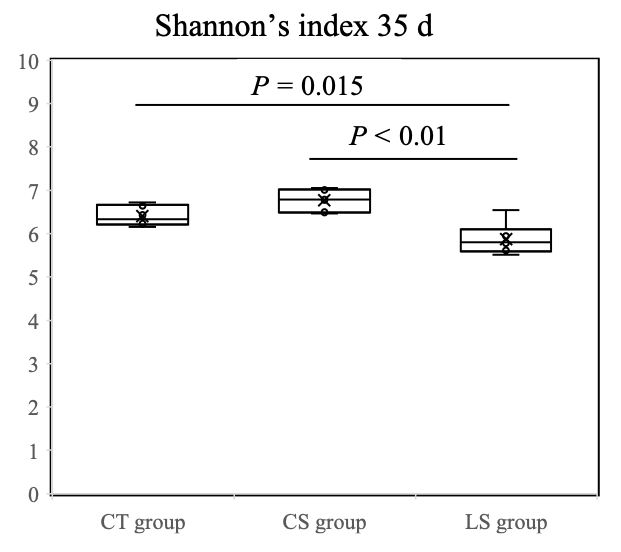
Supplemented Figure 5C**

**
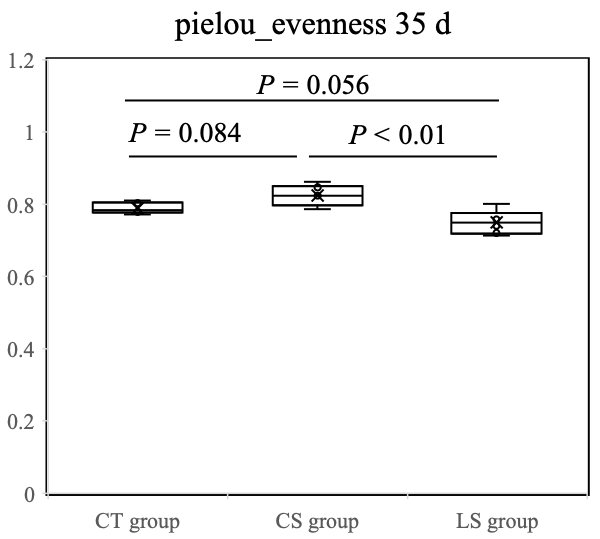
**

**Supplemented Figure 6A**

**
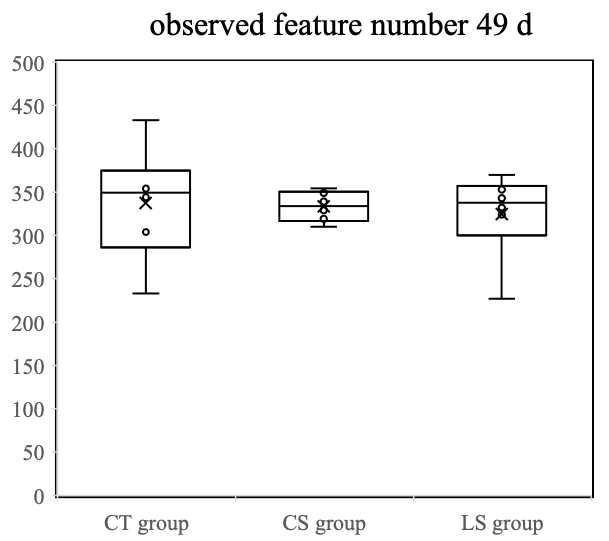
**

**Supplemented Figure 6B**

**
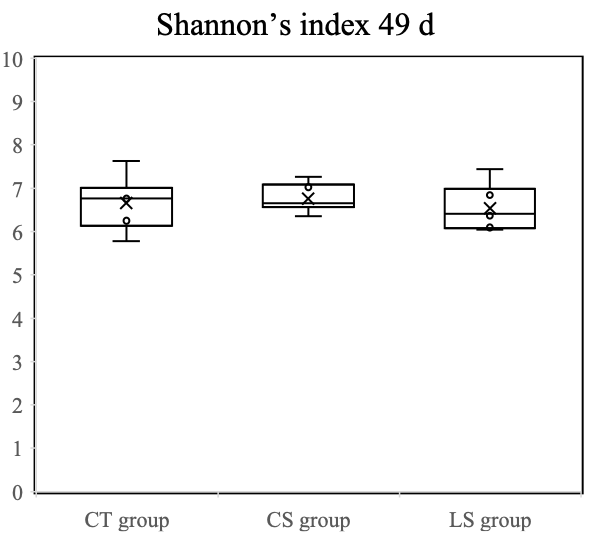
**

**Supplemented Figure 6C**

**
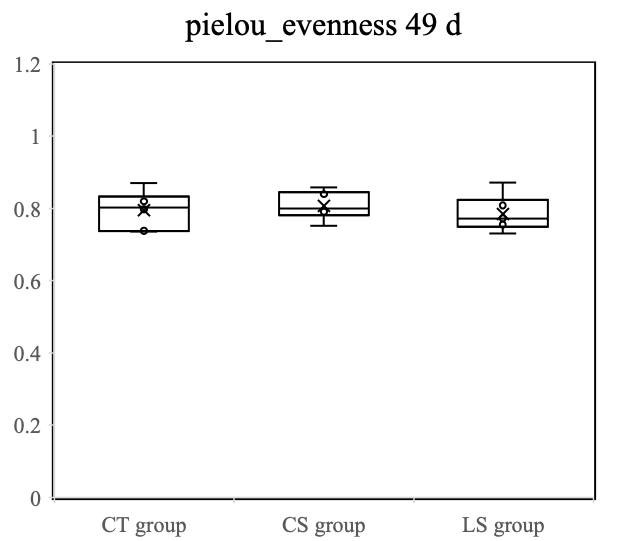
**

**Supplemented Figure** 7

**
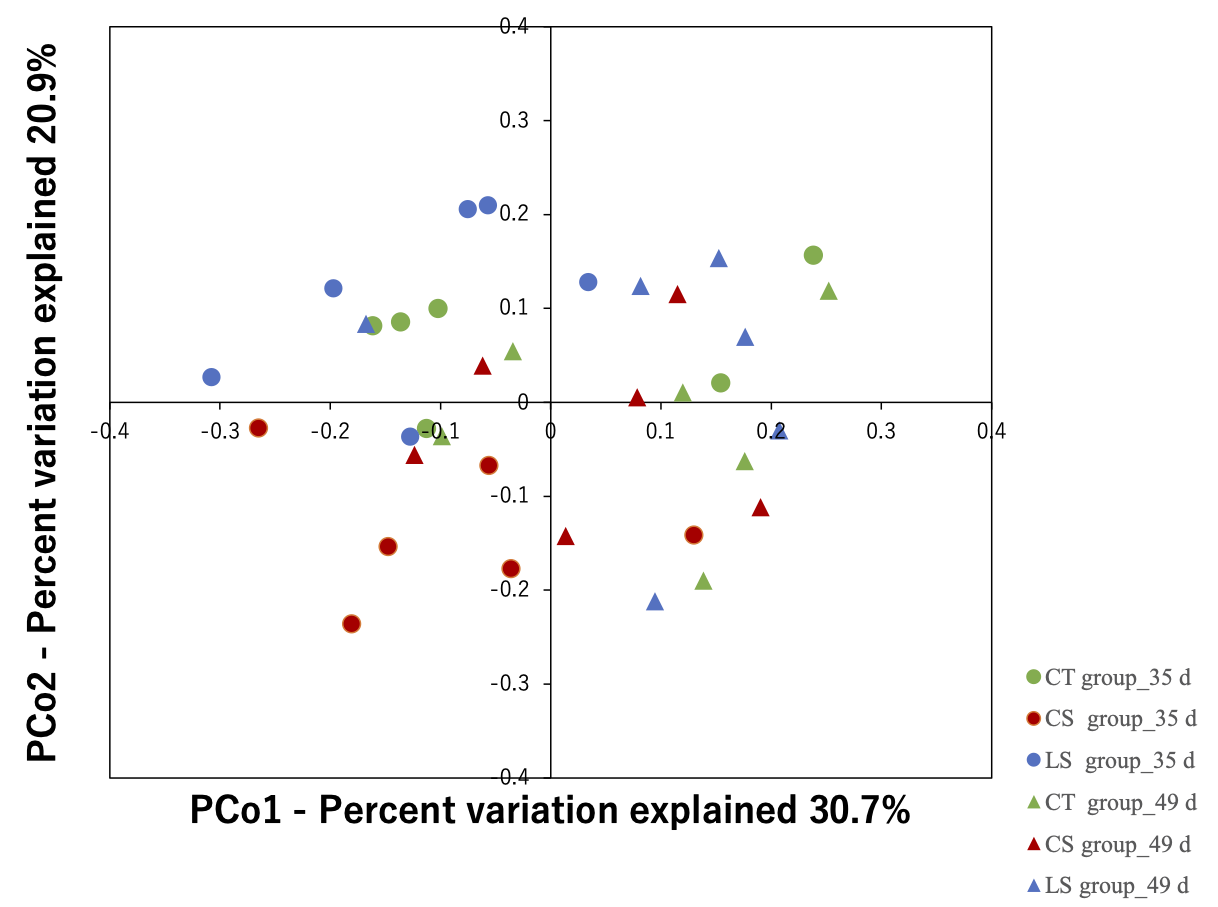
**

**Supplemented Table 1**

| **Ingredient** | **Content, %** | **Chemical Composition** | **Content** |
| --- | --- | --- | --- |
| Corn | 34.45 | CP, % | 23.24 |
| Defatted milk powder | 18 | DE, Mcal/kg | 3.7 |
| Fatty powder | 6.2 | TDN, % | 84.79 |
| Sugar | 10 | Ca, % | 0.81 |
| Soybean meal | 25 | NpP, % | 0.45 |
| Fish meal, CP 65% | 4.5 | Na, % | 0.26 |
| Calcium diphosphate | 0.2 | Cl, % | 0.36 |
| Calcium carbonate | 0.65 | K, % | 0.99 |
| Salt | 0.2 | Mg, % | 0.14 |
| B vitamins^1^ | 0.15 | Fe, mg/kg | 182.18 |
| Vitamins A, D and E^2^ | 0.1 | Zn, mg/kg | 105.32 |
| Trace minerals^3^ | 0.15 | Mn, mg/kg | 87.51 |
| L-Lysine hydrochloride | 0.06 | Cu, mg/kg | 125.29 |
| DL-Methionine | 0.09 | I, mg/kg | 1.95 |
| L-Threonine | 0.03 | Se, mg/kg | 0.3 |
| Copper sulphate | 0.21 | Vitamin A, IU/kg | 100051.62 |
| Vitamin K_3_ | 0.01 | Vitamin D, IU/kg | 2000 |
| Total | 100 | Vitamin E, IU/kg | 20.04 |
|  |  | Vitamin K, IU/kg | 0.57 |
|  |  | Thiamine, mg/kg | 5.15 |
|  |  | Riboflavin, mg/kg | 15.38 |
|  |  | Pantothenic acid, mg/kg | 27.83 |
|  |  | Nicotinic acid, mg/kg | 25.63 |
|  |  | Vitamin B_6_, mg/kg | 5.93 |
|  |  | Choline, mg/kg | 1204.8 |
|  |  | Vitamin B_12_, μg/kg | 21.88 |
|  |  | Biotin, mg/kg | 0.16 |
|  |  | Folic acid, mg/kg | 0.36 |

**Supplemented Figure 1** Spearman correlation analysis between ADG and nutritional status of weaned piglets. (A) Correlation between ADG and plasma amino acids. (B) Correlation between ADG and fecal acetate. The strength (Spearman’s ρ value) and significance of correlations are shown

**Supplemented Figure 2** Concentrations of intestinal (duodenum, jejunum, ileum) mucosal sIgA of weaned piglets at 49 d. The mean and SEM are shown.

**Supplemented Figure 3** Predominant taxa by relative abundance detected in feces at the phylum level. (**a**) Relative abundance of composition of gut microbiota at 35 d. (**b**) Relative abundance of composition of gut microbiota at 49 d.

**Supplemented Figure 4** Predominant taxa by relative abundance detected in feces at the genus level. (**a**) Relative abundance of composition of gut microbiota at 35 d. (**b**) Relative abundance of composition of gut microbiota at 49 d.

**Supplemented Figure 5** Measures of ɑ-diversity of gut microbiota of weaned piglets at 35 d. (A) The box plots of observed feature number of the gut microbiota of weaned piglets at 35 d. (B) The box plots of Shannon’s index of the gut microbiota of weaned piglets at 35 d. (C) The box plots of pielou_evenness of the gut microbiota of weaned piglets at 35 d.

**Supplemented Figure 6** Measures of ɑ-diversity of gut microbiota of weaned piglets at 49 d. (A) The box plots of observed feature number of the gut microbiota of weaned piglets at 49 d. (B) The box plots of Shannon’s index of the gut microbiota of weaned piglets at 49 d. (C) The box plots of pielou_evenness of the gut microbiota of weaned piglets at 49 d.

**Supplemented Figure 7** Measures of ß-diversity of gut microbiota of weaned piglets at 35 d and 49 d. Principal coordinates analysis plot based on the weighted UniFrac distances for gut microbiota composition in weaned piglets in each group at 35 d and 49 d.

**Supplemented Table 1** Ingredients and composition of feed for piglets

**Abbreviation**: CP, crude protein; DE, digestible energy; TDN, total digestible nutrients; NpP, non-phytate phosphorus

Per kilogram thiamine mononitrate: 1,000mg, riboflavin: 7,000mg, pyridoxine hydrochloride: 500mg, calcium pantothenate: 10,900mg, nicotinic acid amide: 6,000mg, choline chloride: 57,600mg, cyanocobalamin: 10mg

^2^ Per kilogram; Vitamin A：10 million IU, Vitamin D_３_： 2 million IU, Tocopherol acetate： 10 g

^3^ Per kilogram; iron sulfate: 50,000 mg, manganese sulfate: 50,000 mg, Zinc carbonate: 50,000 mg, copper sulfate: 10,000 mg, calcium iodate: 1,000 mg
